# Discrimination of mediastinal metastatic lymph nodes in NSCLC based on radiomic features in different phases of CT imaging

**DOI:** 10.1186/s12880-020-0416-3

**Published:** 2020-02-05

**Authors:** Xue Sha, Guanzhong Gong, Qingtao Qiu, Jinghao Duan, Dengwang Li, Yong Yin

**Affiliations:** 1 Shandong Key Laboratory of Medical Physics and Image Processing & Shandong Provincial Engineering and Technical Center of Light Manipulations, School of Physics and Electronics, Shandong Normal University, Jinan, 250358 China; 2 Department of Radiation Oncology, Shandong Cancer Hospital and Institute, Shandong First Medical University and Shandong Academy of Medical Sciences, No.440, Jiyan Road, Jinan, 250117 Shandong China

**Keywords:** Radiomic model, Computed tomography, Mediastinal lymph nodes, Non-small cell lung cancer

## Abstract

**Background:**

We aimed to develop radiomic models based on different phases of computed tomography (CT) imaging and to investigate the efficacy of models for diagnosing mediastinal metastatic lymph nodes (LNs) in non-small cell lung cancer (NSCLC).

**Methods:**

Eighty-six NSCLC patients were enrolled in this study, and we selected 231 mediastinal LNs confirmed by pathology results as the subjects which were divided into training (*n* = 163) and validation cohorts (*n* = 68). The regions of interest (ROIs) were delineated on CT scans in the plain phase, arterial phase and venous phase, respectively. Radiomic features were extracted from the CT images in each phase. A least absolute shrinkage and selection operator (LASSO) algorithm was used to select features, and multivariate logistic regression analysis was used to build models. We constructed six models (orders 1–6) based on the radiomic features of the single- and dual-phase CT images. The performance of the radiomic model was evaluated by the area under the receiver operating characteristic curve (AUC), sensitivity, specificity, accuracy, positive predictive value (PPV) and negative predictive value (NPV).

**Results:**

A total of 846 features were extracted from each ROI, and 10, 9, 5, 2, 2, and 9 features were chosen to develop models 1–6, respectively. All of the models showed excellent discrimination, with AUCs greater than 0.8. The plain CT radiomic model, model 1, yielded the highest AUC, specificity, accuracy and PPV, which were 0.926 and 0.925; 0.860 and 0.769; 0.871 and 0.882; and 0.906 and 0.870 in the training and validation sets, respectively. When the plain and venous phase CT radiomic features were combined with the arterial phase CT images, the sensitivity increased from 0.879 and 0.919 to 0.949 and 0979 and the NPV increased from 0.821 and 0.789 to 0.878 and 0.900 in the training group, respectively.

**Conclusions:**

All of the CT radiomic models based on different phases all showed high accuracy and precision for the diagnosis of LN metastasis (LNM) in NSCLC patients. When combined with arterial phase CT, the sensitivity and NPV of the model was be further improved.

## Background

Lung cancer is one of the most common malignancies and is associated with the highest cancer morbidity and mortality rates worldwide. Non-small cell lung cancer (NSCLC) accounts for approximately 85% of all lung cancers [1]. Lymph node metastasis (LNM) is the most common type of tumor metastasis in NSCLC and remains an essential prognostic factor and guide for adjuvant therapy. In clinical practice, comprehensive treatment that includes surgery, chemotherapy and radiotherapy is the standard treatment for stage I–IIIB NSCLC. However, the scope of lymph node (LN) dissection and the LN areas targeted by radiotherapy remain controversial among different medical centers. Currently, positron emission tomography (PET)/computed tomography (CT) is a relatively accurate imaging technique for the diagnosis of metastatic LNs, with a relatively high specificity for LN staging in patients with NSCLC [2, 3]. However, the low prevalence and high cost of PET/CT equipment limit its clinical application. Additionally, CT has disadvantages for the identification of metastatic LNs, as high rates of false-positive and false-negative results occur when images are analyzed for morphological changes, including size, shape, necrosis, and external capsule invasion [4, 5]. Hence, a great need exists for sensitive and accurate methods to preoperatively assess the status of LNs, which could help to decrease the rate of radical surgery, select appropriate chemotherapy regimens, and delineate the radiotherapy target area.

Due to the emergence of personalized medicine and targeted therapy, the need for quantitative image analysis has increased with the rapid increase in the amount of standard medical data. Radiomics provides promising opportunities in this regard, endowing medical imaging to play an increasingly important role in analyzing tumor heterogeneity [6]. Previous studies have shown that objective and quantitative image features could potentially be used as prognostic or predictive biomarkers [7]. However, most studies have focused on single-phase CT images, which may not obtain the best model from a series of CT images.

Therefore, in the present study, we investigated the accuracy of radiomic and delta radiomic features between CT scans different phases for the preoperative discrimination of metastatic LNs in NSCLC patients to provide the best reference model for the clinical diagnosis of mediastinal lymph nodes.

## Methods

### Patient information

The Institutional Review Board approved the retrospective review of the medical records for this analysis. Participants were selected according to the inclusion and exclusion criteria and were limited to patients treated between January 2015 and June 2018 at our hospital, resulting in a total of 86 patients who were included in this study. The inclusion criteria were as follows: (I) all patients underwent surgical lung resection and systematic LN dissection within 2 weeks after undergoing non-contrast and contrast CT scans; (II) the tumor subtypes and LNs status were confirmed by pathology results; and (III) multiple tumors and other manifestations were absent. Due to the high cost and low prevalence of PET/CT, it was not required for eligibility in preoperative examinations for this retrospective study. The exclusion criteria were as follows: (I) clinical data were incomplete, or statistical analysis could not be performed; (II) patients received treatments before the scans were performed; (III) poor image quality affected the quantitative analysis; and (IV) CT images were reconstructed using different algorithms, thicknesses, or equipment.

Then the enrolled patients were divided into two independent cohorts: 61 patients treated between January 2015 and June 2017 constituted the training cohort, and 25 patients treated between July 2017 and June 2018 constituted the validation cohort. Tumor subtypes and lymph node status were proven by pathological results, and clinical factors including gender and stage were derived from medical records. Disease stage was evaluated according to the TNM Classification of Malignant Tumors, 7th Edition.

### CT image acquisition

All patients underwent routine and enhanced CT scanning, and a Philips scanner (Holland, CT LightSpeed 16) was used with the following imaging protocol: tube voltage 120 kV, cube current 300 mA, thickness 2 mm and in-plane resolution 0.97 × 0.97. The contrast medium was injected into the elbow vein at an injection rate of 2.3~3.0 ml/second, and the maximum dose was 100 ml. An arterial phase scan was performed 25 to 30 s after contrast medium injection, and a venous phase scan was performed 90 s later. Plain, arterial and venous phase images were obtained. All images were exported in the Digital Imaging and Communications in Medicine (DICOM) format for image feature extraction.

### Radiomics workflow

The radiomics workflow included: (1) image segmentation, (2) feature extraction, (3) feature selection, and (4) predictive model building.

### Lesion segmentation

**Fig. 1:**
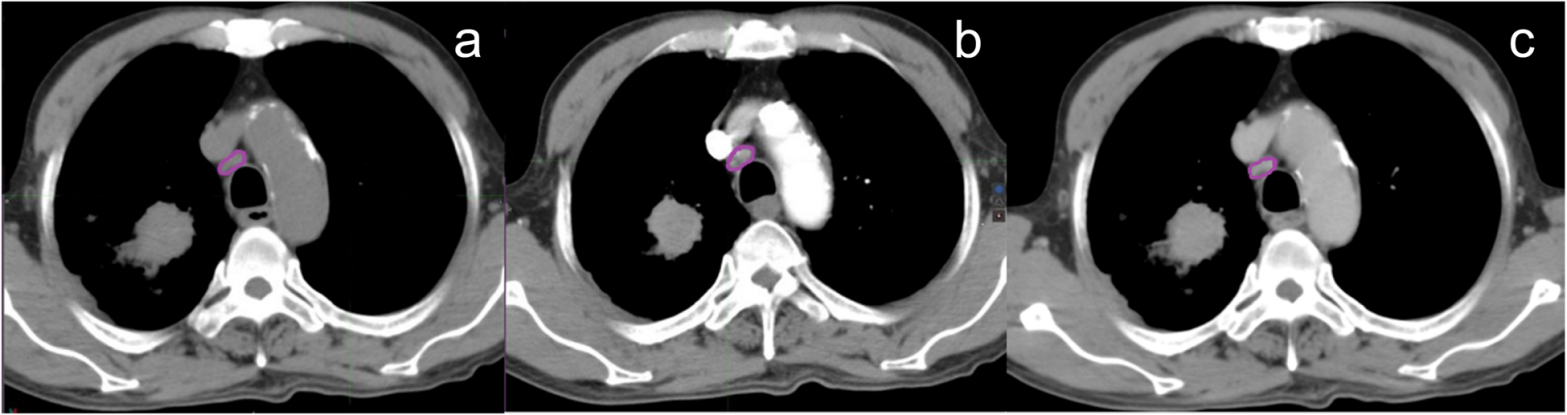
CT images from a 56-year-old man with mediastinal LNM confirmed by pathology. Panels **a**, **b** and **c** show the ROIs on plain, arterial and venous phase CT images, respectively

We performed manual segmentation on arterial phase CT images using MIM Maestro version 6.8.2 (MIM software, Cleveland, OH), and pathologically confirmed LNs were defined as regions of interest (ROIs). Using the arterial phase CT image as the reference, plain and venous phase CT images were corrected by the nonrigid registration method, and the contouring results were mapped to the plain and venous phase images, respectively. The target images were delineated by two senior radiologists with 20 years of experience in chest CT diagnosis, and differences in the findings were resolved by a third high-ranking radiologist when disputes occurred. Figure 1 shows schematic diagrams of the ROIs on three CT images in different phases.

### Feature extraction

Radiomic features were extracted from LNs using 3D Slicer software, an open-source Python package for the extraction of features from medical images (version 4.6, http://www.slicer.org) [8]. In total, 841 radiomic features were extracted and were organized into two categories: (I) based on original images; and (II) based on wavelet images. Eighteen first-order features derived from the tumor intensity histogram reflected the distribution of the values of individual voxels without concern for spatial relationships. Thirteen shape features provided the geometric tumor volume. Seventy-four texture features described the spatial arrangement of voxels, as calculated from different parent matrices, which included the gray level dependence matrix (GLDM), the gray level cooccurrence matrix (GLCM), the gray level size zone matrix (GLSZM), the gray level run length matrix (GLRLM) and the neighborhood gray-tone difference matrix (NGTDM) [9]. In addition, 736 wavelet features derived from eight filtering modes were obtained.

### Feature selection and radiomic models development

A least absolute shrinkage and selection operator (LASSO) logistic regression algorithm was used to select significant features with nonzero coefficients to develop models. In this study, we constructed six models based on the radiomic features of single-phase imaging and joint two-phase imaging, which included models 1, 2, and 3 (based on the plain, arterial and venous phase radiomic features, respectively), and models 4, 5, and 6 (based on the delta radiomic features between plain and arterial phase imaging, plain and venous phase imaging, and arterial and venous phase imaging, respectively). This process was implemented in R software (version: 3.3.3, https://www.r-project.org). The classification performance of the radiomic models was quantified by the area under the receiver operator characteristic curve (AUC), sensitivity, specificity, accuracy, positive predictive value (PPV) and negative predictive value (NPV) in both the training and validation cohorts.

### Statistical analysis

Data analysis was performed using Statistical Package for Social Sciences (SPSS) software version 23.0 (SPSS, Chicago, IL, USA) and R software (version 3.4.0, https://www.r-project.org). We compared clinical characteristics between the training and verification groups by Wilcoxon ranksum test. *P* values less than 0.05 were considered statistically significant.

## Results

### Characteristics of patients in the training and validation cohorts

The characteristics of patients in both the training and validation cohorts were displayed in Table 1. A total of 231 LNs which has surgical-pathologic information were examined from 86 patients, including 58 males and 28 females aged 35–84 years. In the training cohort, 60.7% (99/163) of LNs were pathologically positive, and 39.3% (64/163) of LNs were pathologically negative. In the validation cohort, 61.8% (42/68) of LNs were pathologically positive, and 38.2% (26/68) of LNs were pathologically negative. No significant differences were observed in two cohorts, with *P*-value ranging from 0.139 to 0.885.

**Table 1:** Characteristics of patients in the training and validation cohorts

Characteristic	Training Cohort	Validation Cohort	*P*-value
Gender, n(%)			0.570
male	40 (65.6)	18 (72.0)	
female	21 (34.4)	7 (28.0)	
Age (years)			0.139
Mean	58.64	60.25	
Range	35–84	42–78	
T stage, n(%)			0.818
T1	16 (26.2)	6 (24.0)	
T2	28 (45.9)	14 (56.0)	
T3	7 (11.5)	1(4.0)	
T4	10 (16.4)	4 (16.0)	
N stage, n(%)			0.123
N0	9(14.8)	7 (28.0)	
N1	6 (9.8)	2 (8.0)	
N2	19 (31.1)	9 (36.0)	
N3	27 (44.3)	7 (28.0)	
M stage, n(%)			0.576
M0	35 (57.4)	16 (64.0)	
M1	26 (42.6)	9 (36.0)	
Pathological subtype, n(%)			0.394
Adenocarcinoma	36 (59.0)	17 (68)	
Squamous cell carcinoma	23 (37.7)	8 (32)	
Large cell lung cancer	2 (3.3)	0 (0)	
Lymph nodes status, n(%)			0.885
Positive	99 (60.7)	42 (61.8)	
Negative	64 (39.3)	26 (38.2)	

### Selection of features and the construction of radiomic models

**Fig. 2:**
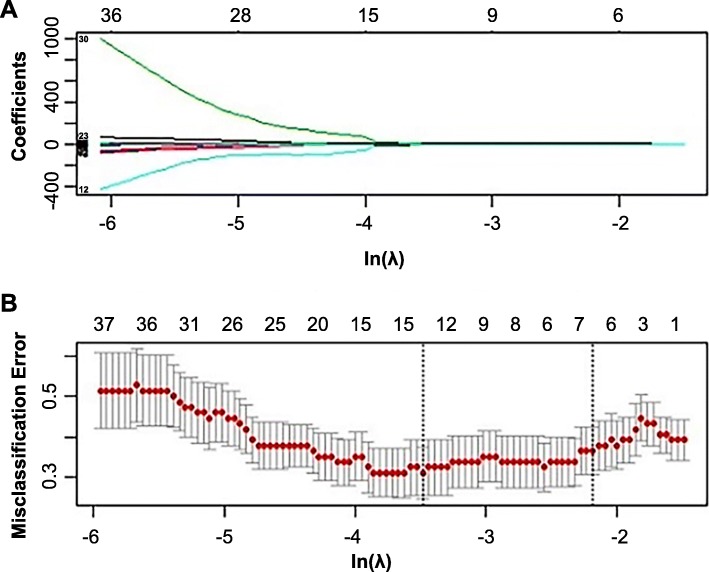
The feature selection process. **a** LASSO coefficient profiles of the 841 features. **b** Tuning parameters (λ) selected in the LASSO model were used for applied 10-fold cross-validation with the minimum criteria. The Y-axis indicates misclassification errors, and the lower X-axis indicates the average deviance ln(λ) values, which were − 2.19, − 2.44, − 2.14, − 1.61, − 1.75, and − 2.61 in models 1–6, respectively. The vertical lines through the red dots show the upper and lower limits of the deviances. Dotted vertical lines were drawn at the optimal values using the minimum criteria with 1 standard error (the 1-SE criteria). Numbers along the upper X-axis represent the average number of predictors

**Fig. 3:**
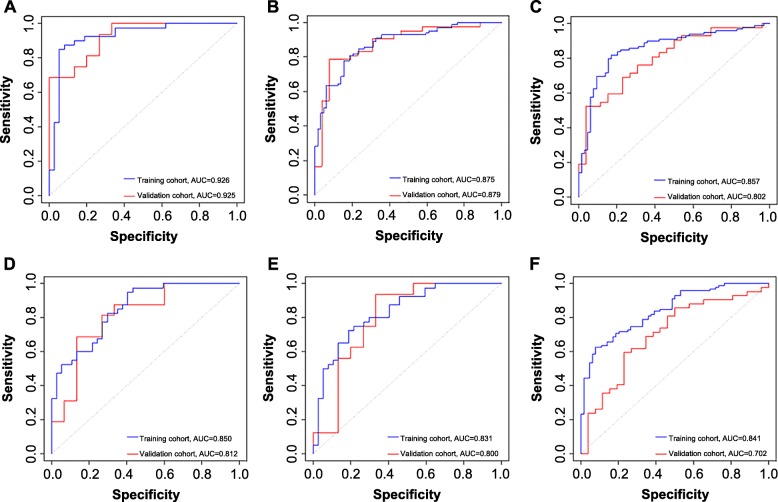
ROC curves of the radiomic models. Panels **a**-**f** correspond to models 1–6, respectively

A total of 841 features were extracted from each phase CT image of the training cohort. We screened these features and chose 10, 9, 5, 2, 2, and 9 features that had nonzero coefficients as potential predictors using the LASSO logistic regression model. The weighted values of the nonzero characteristics selected to develop models were summarized in an Additional file 1. Figure 2 depicted the process of feature selection. The radiomic models all showed a favorable predictive efficacy for identifying mediastinal LNM in NSCLC patients, with AUC values higher than 0.830 in the training cohort (Fig. 3).

### Analysis of models based on the single- and joint-phase CT

Based on single-phase CT images and joint-phase CT images, we constructed six models in this study. As shown in Table 2, model 1 yielded the highest AUC, specificity, accuracy and PPV, which were 0.926 and 0.925; 0.860 and 0.769; 0.871 and 0.882; and 0.906 and 0.870 in the training and validation sets, respectively. However, the sensitivity and NPV of model 2 were higher than those of the other single-phase models. We observed that the AUC value of model 4–6 was lower than that of model 1–3, but the sensitivity and NPV of the training group were significantly higher. Compared with those of model 1, the sensitivity and NPV of model 4 increased from 0.879 and 0.821 to 0.949 and 0.878, respectively. Moreover, compared with those of model 3, the sensitivity and NPV of model 6 increased from 0.919 and 0.789 to 0.979 and 0.900, respectively. The sensitivity, specificity, accuracy, NPV, and PPV of each model are listed in Table 2.

**Table 2:** Efficacy of models for identifying mediastinal LNM in the training and validation groups

Model	Group	AUC	SEN	SPE	ACC	PPV	NPV
1	training	0.926	0.879	0.860	0.871	0.906	0.821
	validation	0.925	0.952	0.769	0.882	0.870	0.909
2	training	0.875	0.929	0.609	0.804	0.786	0.848
	validation	0.876	0.976	0.423	0.765	0.732	0.917
3	training	0.857	0.919	0.469	0.742	0.728	0.789
	validation	0.802	0.905	0.500	0.750	0.745	0.765
4	training	0.850	0.949	0.563	0.798	0.770	0.878
	validation	0.813	0.952	0.423	0.750	0.727	0.846
5	training	0.831	0.879	0.594	0.767	0.770	0.760
	validation	0.800	0.952	0.615	0.824	0.889	0.889
6	training	0.841	0.979	0.281	0.706	0.678	0.900
	validation	0.702	0.928	0.192	0.647	0.650	0.625

*SEN* sensitivity, *SPE* specificity, *ACC* accuracy, *PPV* positive predictive value, *NPV* negative predictive value

## Discussion

The International Association for the Study of Lung Cancer (IASLC) showed that, based on a newly established large database, the 5-year survival rates for patients with LNM ranged from 26 to 53% [10]. The systematic dissection of LNs in lung cancer patients has been widely accepted, but the extent of LN dissection has remained a matter of debate due to the precise assessment of metastatic LNs [11, 12]. LNM is an important factor that affects tumor and LN staging. Therefore, the noninvasive preoperative evaluation of the LN status is crucial for determining the lung cancer stage, surgical plan, and prognosis [13].

Currently, CT are the most routinely used noninvasive methods for the clinical diagnosis of LNs. The international standard for the diagnosis of metastatic LNs by CT in lung cancer is a short-axis LN diameter larger than 10 mm. However, due to the single diagnostic criterion, the accuracy of the diagnosis is limited to some extent. Also, PET/CT is a noninvasive method for staging cancer that has been increasingly employed by multidisciplinary lung cancer teams. Many studies have reviewed the diagnostic performance of PET/CT for LN staging in patients with NSCLC [14–16]. A systematic review showed that the summary sensitivity and specificity estimates for a maximum standard uptake volume (SUVmax) ≥2.5, which is the PET/CT positivity criterion, were 81.3 and 79.4%, respectively [17]. However, the low prevalence and high cost of PET/CT equipment have resulted in it being less commonly used than CT alone in preoperative examinations. If the accuracy of CT in the diagnosis of LNs could be improved, it would provide more important clinical guidance for identifying the radiotherapy targets and surgical range.

Recently, the development of radiomics has enabled medical images to be converted into high-throughput quantitative data, providing information that can be explored and used to guide clinical decision-making. In contrast to subjective descriptions of the volume and shape of lesions, radiomic features can more comprehensively describe the state of lesions, overcoming the disadvantages of traditional diagnostic methods [18–20]. Therefore, radiomics is expected to improve the accuracy of diagnosis based on CT images. Moreover, studies have demonstrated the feasibility of using radiomic features to predict LNM in rectal, breast and esophageal cancers, providing theoretical support for this study [21–23].

In the present study, we constructed radiomic models based on pathological diagnostic results to facilitate the preoperative identification of metastatic LNs in NSCLC patients. The results showed that the diagnostic models based on different phases all exhibited favorable discrimination (AUC values greater than 0.8, a maximum sensitivity of 97.9%, and a maximum specificity of 86.0%), and model 1 (plain CT) yielded the highest AUC, specificity, accuracy and PPV. The underlying reason for the better performance on non-contrast images may be that the biological heterogeneity within the LNs that can be described by radiomic features may be confounded by the intravenous injected contrast material, which may then lead to worse discrimination between malignant and benign LNs due to the existing intratumoral contrast material. On the other side, the result of this stduty showed that more texture features (10 texture features) were selected from non-contrast CT than contrast-enhanced CT (9 or 5 texture features), and the texture features from plain CT scan were found to be more significant in discriminating mediastinal metastatic lymph nodes. Moerover, previous researches have confirmed this interesting finding. He et al. [24] evaluated the effects of contrast-enhancement on the diagnostic performance of radiomics signatures in solitary pulmonary nodules (SPNs), which indicated that contrast-enhancement can affect the diagnostic performance of radiomics signatures in SPNs and that non-contrast CT is more informative. Similarly, Sui et al. [25] confirmed that the radiomics features of the non-contrast CT have a better predictive performance than those of contrast CT in anterior mediastinal lesion risk grading. In the research of classifying mediastinal LNM of NSCLC from ^18^F-FDG PET/CT images, Yao et al. [26] summarized the diagnostic results from 22 research centers and found that the overall sensitivity and specificity were 0.66 and 0.82, respectively. In addition, another study showed that the sensitivity and specificity of CT for the diagnosis of mediastinal LNM were 0.79 and 0.72, respectively [27]. Compared to those published studies, the methods proposed in our study have the advantages of being quantitative and reproducible, with a higher sensitivity and specificity than the previously reported methods.

Moreover, we not only extracted radiomic features from plain, arterial, and venous phase CT images but also calculated delta radiomic feature values between different phase CT images in different phases. The arterial phase mainly reflects the tissue perfusion of the tumor, and the venous phase mainly reflects the clearing of the tissue blood flow, which is also an important imaging feature of tumor metastasis [28]. Dynamic CT texture analysis can assess temporal changes in tumor heterogeneity after the administration of contrast material and could provide another dimension of physiologic tumor assessment [29]. The sensitivity and NPV of the model were significantly better when combined with arterial phase CT in our study, which may have been because temporal changes in texture features can potentially provide diagnostic and prognostic information and canincrease the utility of contrast-enhanced CT. In clinical practice, for NSCLC patients treated with neoadjuvant therapy and routine radical surgery, false-positive LNs will not result in insufficient treatment or lead to treatment delay. However, the higher NPV of this approach means that negative LNs will be more accurately identified, which may change the clinical treatment plan [23]. These findings suggest that the accuracy of models can be improved when combined with dual-phase radiomic features in future clinical applications.

This method of integrating a large number of features in CT images that cannot be recognized or distinguished by the human eye has high accuracy and sensitivity for diagnosing mediastinal LNM in NSCLC patients and is expected to improve the efficacy of treatments for NSCLC. However, there are still some limitations of this study. First, the data used in this study were obtained from the same center. Second, the diagnostic capacity of combined clinical and quantitative imaging features could not be evaluated. Third, a minority of patients in our study underwent both CT and PET/CT before surgery because of the high cost of PET/CT and the high radiation exposure. In addition, this study only demonstrated the feasibility of discriminating mediastinal LNs. And we will explore the differences in diagnostic performance of mediastinal, hilar and intralobar LNM since adequate LNs were collected in the future. In summary, all of the CT radiomic models based on different phases all showed high accuracy and precision for the diagnosis of LNM in NSCLC patients. The combination of plain and venous phase CT scans with arterial phase CT radiomic features can further improve the sensitivity and NPV.

## Conclusions

Our study demonstrated the superiority of CT radiomic analysis for diagnosing mediastinal metastatic LNs in NSCLC patients and presents the difference between radiomic models of non-contrast and contrast-enhanced CT. All of the radiomic models based on different CT phases showed high accuracy and precision, and the sensitivity and NPV was be further improved when combined with arterial phase CT. Therefore, we believe it is a promising and applicable alternative approach for discriminating LNs of NSCLC with the advantage of quantitative, non-invasive and low cost, which may serve as a complementary tool to facilitate making clinical decisions.

## Supplementary information

**Additional file 1: Table S1.** Selected features of non-zero weight in single-phase model. **Table S2.** Selected features of non-zero weight in two-phase model.

## Data Availability

The datasets generated and analyzed during the current study are available from the corresponding author on reasonable request (yinyongsd@126.com).

## Abbreviations

ACC: Accuracy
AUC: Area under the receiver operator characteristic curve
GLCM: Gray level co-occurrence matrix
GLDM: Gray level dependence matrix
GLRLM: Gray level run length matrix
GLSZM: Gray level size zone matrix
LASSO: Least absolute shrinkage and selection operator
LNM: Lymph node metastasis
NGTDM: Neighborhood gray-tone difference matrix
NPV: Negative predictive value
NSCLC: Non-small cell lung cancer
PET/CT: Positron emission tomography/computed tomography
PPV: Positive predictive value
ROIs: Regions of interest
SE: Standard error
SEN: Sensitivity
SPE: Specificity
