# Supplementary material for: Discrimination of mediastinal metastatic lymph nodes in NSCLC based on radiomic features in different phases of CT imaging
Source: BMC Med Imaging. 2020 Feb 5;20:12. doi: 10.1186/s12880-020-0416-3 (PMC7003415; doi:10.1186/s12880-020-0416-3)
Supplement: Supplementary file 1 — Additional file 1: Table S1. Selected features of non-zero weight in single-phase model. Table S2. Selected features of non-zero weight in two-phase model. [file 12880_2020_416_MOESM1_ESM.docx]

**Table S1.** Selected features of non-zero weight in single-phase model

| Model 1 | | Model 2 | | Model 3 | |
| --- | --- | --- | --- | --- | --- |
| Feature name | Weight | Feature name | Weight | Feature name | Weight |
| Inverse Variance | 3.934 | Inverse Variance | 1.927 | 10 Percentile | 0.005 |
| Idn | 2.576 | Zone Entropy | 0.200 | Median | 0.002 |
| Small Area Low Gray Level Emphasis | 1.106 | Median | 0.033 | Skewness | -0.035 |
| Small Area Emphasis | 0.784 | 10Percentile | 0.005 | Zone Percentage | -0.225 |
| Median | 0.020 | Median | 0.002 | Surface Volume Ratio | -2.707 |
| 10Percentile | 0.006 | Strength | -0.082 | —— | —— |
| Skewness | -0.099 | Maximum Probability | -0.352 | —— | —— |
| Dependence Non Uniformity Normalized | -0.604 | Id | -2.039 | —— | —— |
| Coarseness | -4.026 | Surface Volume Ratio | -3.397 | —— | —— |
| Zone Percentage | -8.328 | —— | —— | —— | —— |

**Table S2.** Selected features of non-zero weight in two-phase model

| Model 4 | | Model 5 | | Model 6 | |
| --- | --- | --- | --- | --- | --- |
| Feature name | Weight | Feature name | Weight | Feature name | Weight |
| Dependence Entropy | -0.459 | Small Area Low Gray Level Emphasis | 0.317 | Gray Level Non Uniformity Normalized | 9.484 |
| Root Mean Squared | -0.010 | Root Mean Squared | -0.006 | Maximum Probability | 1.459 |
| —— | —— | —— | —— | Difference Average | 0.423 |
| —— | —— | —— | —— | Skewness | 0.068 |
| —— | —— | —— | —— | Robust Mean Absolute Deviation | 0.029 |
| —— | —— | —— | —— | Large Dependence Emphasis | -0.015 |
| —— | —— | —— | —— | Inverse Variance | -0.669 |
| —— | —— | —— | —— | Strength | -0.817 |
| —— | —— | —— | —— | Joint Energy | -4.252 |
